# Recent developments in muscle synergy analysis in young people with neurodevelopmental diseases: A Systematic Review

**DOI:** 10.3389/fbioe.2023.1145937

**Published:** 2023-04-27

**Authors:** Giulia Beltrame, Alessandro Scano, Giorgia Marino, Andrea Peccati, Lorenzo Molinari Tosatti, Nicola Portinaro

**Affiliations:** ^1^ Residency Program in Orthopedics and Traumatology, Universitá degli Studi di Milano, Milan, Italy; ^2^ Institute of Intelligent Industrial Systems and Technologies for Advanced Manufacturing (STIIMA), Italian Council of National Research (CNR), Milan, Italy; ^3^ Physiotherapy Unit, Humanitas Clinical and Research Center—IRCCS, Milan, Italy; ^4^ Department of Pediatric Surgery, Fondazione IRCCS Ca’ Granda, Ospedale Maggiore Policlinico, Milan, Italy

**Keywords:** muscle synergies, neurodevelopmental diseases, cerebral palsy, biomarkers, rehabilitation, review

## Abstract

The central nervous system simplifies motor control by sending motor commands activating groups of muscles, known as synergies. Physiological locomotion can be described as a coordinated recruitment of four to five muscle synergies. The first studies on muscle synergies in patients affected by neurological diseases were on stroke survivors. They showed that synergies can be used as biomarkers for motor impairment as they vary in patients with respect to healthy people. Likewise, muscle synergy analysis has been applied to developmental diseases (DD). The need for a comprehensive view of the present findings is crucial for comparing results achieved so far and promote future directions in the field. In the present review, we screened three scientific databases and selected thirty-six papers investigating muscle synergies extracted from locomotion in children affected by DD. Thirty-one articles investigate how cerebral palsy (CP) influences motor control, the currently exploited method in studying motor control in CP and finally the effects of treatments in these patients in terms of synergies and biomechanics; two articles investigate how muscle synergies vary in Duchenne muscular dystrophy (DMD), and three other articles assess other developmental pathologies, such as chronic and acute neuropathic pain. For CP, most of the studies demonstrate that the number of synergies is lower and that the synergy composition varies in the affected children with respect to normal controls. Still, the predictability of treatment’s effects and the etiology of muscle synergy variation are open questions, as it has been reported that treatments minimally modify synergies, even if they improve biomechanics. The application of different algorithms in extracting synergies might bring about more subtle differences. Considering DMD, no correlation was found between non-neural muscle weakness and muscle modules’ variation, while in chronic pain a decreased number of synergies was observed as a possible consequence of plastic adaptations. Even if the potential of the synergistic approach for clinical and rehabilitation practices is recognized, there is not full consensus on protocols nor widely accepted guidelines for the systematic clinical adoption of the method in DD. We critically commented on the current findings, on the methodological issues and the relative open points, and on the clinical impact of muscle synergies in neurodevelopmental diseases to fill the gap for applying the method in clinical practice.

## Introduction

Locomotion requires the central nervous system (CNS) to coordinate a vast number of variables due to the redundant degrees of freedom of the musculoskeletal system. It has been theorized that the central nervous system simplifies the motor control by sending motor commands through a linear combination of motor synergies, each composed of a group of muscles that activates together. Muscle synergies can be considered as an efficient and parsimonious way to control and simplify spatiotemporal patterns of muscle activation by reducing the degrees of freedom to be coordinated (Bizzi et al., 1991; d’Avella and Bizzi, 2005; Cheung et al., 2009; Niemitz, 2010; Zaaimi et al., 2018 **).**

Muscle synergy analyses have been applied to several fields of research. Pioneeristic studies on stroke survivors have shown synergistic control in both the affected and unaffected upper limb (Cheung et al., 2009). In severely impaired patient merged and fractionated motor modules were found; in mildly affected patients, synergy muscle weightings were similar in composition between cases and controls, but the activation timing profiles may change, showing more variability or overlapping of temporal coefficients across modules in the control group (Cheung et al., 2012).

Adult locomotion can be described as the subsequent activation of four to five muscle synergies (Ivanenko et al., 2004; Cappellini et al., 2006). Differences in modular organization have an impact on functional consequences as impairment in self-selected walking speed, propulsive asymmetry and step length asymmetry (Clark et al., 2010). The biomechanical implications found in post stroke patients associated with synergies’ variation impose to know more about how muscle synergies vary also in other neurological pathologies, especially in developmental diseases (DD). In clinical settings, a better understanding on how muscles synergies vary among healthy children and children affected by cerebral palsy as well as by other DD would provide a deeper insight on the possible therapeutical options and their outcomes.

Cerebral palsy (CP) is the most common cause of chronic disability in childhood, occurring in about 2–2.5 per 1,000 live births (Jan 2006). CP is the clinical presentation of a wide variety of cerebral cortical and sub-cortical lesions occurring in the pre-natal, peri-natal and post-natal periods, up to the first 2 years of life (Jan 2006). It represents a group of permanent motor and cognitive disorders due to a non-progressive damage to the developing brain, without a univocal etiology, eventually resulting in inabilities to selectively control muscles. From a clinical point of view, the most important classification relates motor abilities and functional limitations with the Gross Motor Function Classification System (GMFCS), which is a scale of five levels, ranging from walking without limitations (level I) to being on a wheelchair (level V) (Palisano et al., 1997; Rosenbaum et al., 2008). The therapeutical options directly correlate to the level of disability, ranging from physical therapy, drugs for spasticity, orthopedic and neurosurgical interventions. Many patients require a combination of the previously mentioned therapies. The therapeutic challenge relies on providing an individualized treatment plan which is patient-oriented, goal-oriented and cost-effective (Pavone et al., 2016). This is the reason why there has been a growing interest in muscle synergy analysis which has the potential to provide a quantitative method for gait assessment in clinics. The current knowledge on the topic demonstrates that the number of muscle synergies is lower in CP than in healthy subjects and it decreases when the GMCSF level increases (Kim et al., 2021). The most affected synergies are those related to the knee-foot and the knee-hip couples, which result to be impaired in most patients (Thelen et al., 2003; Czupryna and Nowotny, 2012). In addition, children affected by CP show a higher stride-to-stride variability, which is not observed in healthy controls (Kim et al., 2018; Yu et al., 2019; Bekius et al., 2021). Bekius and others have recently summarized the evolution in the use of muscle synergies as biomarkers for evaluating locomotion in children affected by CP (Bekius et al., 2020). However, many articles have been published since then. Furthermore, even if CP is the most common cause of gait impairment in children, other DD affect locomotion and the evaluation of other pathologies presenting different pathogeneses might help in defining when muscle synergy analysis is useful in clinical practice.

Duchenne muscular dystrophy (DMD) is an X-linked degenerative muscular disease, affecting 1 in 3,500–6,000 male births. The absence of the protein dystrophin is responsible for the infiltration of the muscular tissue with non-contractile fibrofatty tissue (Sussman, 2002). The typical clinical sign is muscular weakness which influences walking ability. According to the few studies focusing on the relation between muscle weakness and motor control, muscle synergies do not change significantly between the DMD group and the control group (Goudriaan et al.,2018; Vandekerckhove et al., 2020).

Other pathologies that affect gait have been analyzed by exploiting muscle synergies analysis. Hemophilic arthropathy is a systemic arthropathy mostly caused by hemophilia and characterized by repetitive hemarthroses as well as progressive joint involvement (Cruz-Montecinos et al., 2019). Affected children show a higher total variance accounted for (tVAF) of muscle synergies compared to the control group (Cruz-Montecinos et al., 2019). Toddlers affected by Down syndrome need to compensate the increased joint laxity and the decreased muscle tone by using different strategies of muscle synergies, in terms of rhythmicity of their muscle bursts (Chang et al., 2009). Spinal cord injuries impose the coactivation of flexors and extensors (Fox et al., 2013).

In the present review, we collected the state of art on muscles synergy analysis in DD, with a special interest in CP given the increasing number of published papers on the topic, integrating the previous findings with the latest pieces of evidence, focusing on the new trends on synergies analysis and extraction. Novel work not only brought further pieces of evidence regarding pathological gait, but also proposed new techniques, including the use of recently released algorithms or multi-modal approaches that may shed light on mechanisms of recovery that have not been fully understood yet. Regarding the methodology of synergy extraction, in the present review we focused on all data processing steps such as normalization and data structure, which are of great interest since the technical methodology may impact the clinical interpretation. Many open points have been lately suggested to improve the field of muscle synergies analysis in DD, such as the need of standardizing the synergy extraction methods, the need of expanding the use of novel computational approaches that increase interpretation power, as well as the need of understanding the etiology of muscle synergy variation and the prediction of the effects of rehabilitation treatments on synergies. To better understand what is known on muscle synergy analysis in other DD, we also extended the investigation to the role of muscle synergies in other developmental pathologies, other than CP. With these aims, we performed a systematic review, screening three main databases to provide a comprehensive summary of the current knowledge on muscles synergies analysis in DD.

## 2 Materials and methods

This review was conducted according to the international guidelines established by PRISMA (Preferred Reporting Items for Systematic Reviews and Meta-Analyses) **(** Liberati et al., 2009 **)**.

### 2.1 Research questions

The papers that have been considered for this review exploit multi-channel EMG signals recorded from lower limb muscles during walking in TD (typically developed) children and children affected by DD. The main research question of the review is to investigate how muscle synergies vary between typically developed children and children affected by DD during locomotion. Particularly, reviewers are interested in investigating the differences in muscle synergies between TD and CP groups. A focus is also dedicated to how rehabilitative treatments and interventions influence muscle synergies in CP to investigate the effects of longitudinal studies.

Reviewers intend also to emphasize which processing steps have been used to extract muscle synergies in DD, in order to evaluate how reliable is the comparison between studies, and which steps can be made to improve the understanding of the results and the interpretative power of the recorded data.

### 2.2 Bibliographic research criteria

With the above-mentioned aims, a collection of articles was obtained by screening the databases PubMed, Scopus, and Web of Science (WOS), applying a query based on the following keywords: “CHILDREN”, “YOUNG ADULT”, “ADOLESCENT”, “MUSCLE SYNERGY”, “LOCOMOTION”, “WALK”, “LOWER LIMB”, “DEAMBULATION”, “GAIT”, and excluding “RUN”.

The formal logical query was [(CHILDREN) OR (CHILD) OR (YOUNG ADULT) OR (ADOLESCENT)] AND [(MUSCLE SYNERGY) OR (MUSCLE SYNERGIES)] AND [(LOCOMOTION) OR (WALK) OR (LOWER LIMB) OR (DEAMBULATION) OR (GAIT)] AND NOT (RUN). Databases were searched up to 5 January 2023.

### 2.3 Eligibility criteria

To be included in the final review, the screened papers had to satisfy the following criteria: (A) to include the specified query in the abstract and/or title and/or in the keywords (B) to evaluate muscle synergies with algorithms for synergy extraction (C) to have enrolled volunteers younger than 18 years old (D) to have recruited children affected by developmental diseases (E) to be available in English.

After exclusion of duplicate articles, one of the authors of the review performed a title and abstract screening on the residual articles. Thereafter, the reviewer assessed the eligibility of the remaining articles by a full-text screening. Other authors cross-checked the papers until full agreement was found on all doubts about inclusions. The selected works had to satisfy the inclusion criteria “A and B and C and D and E”.

## 3 Results

### 3.1 Selected papers

**FIGURE 1:**
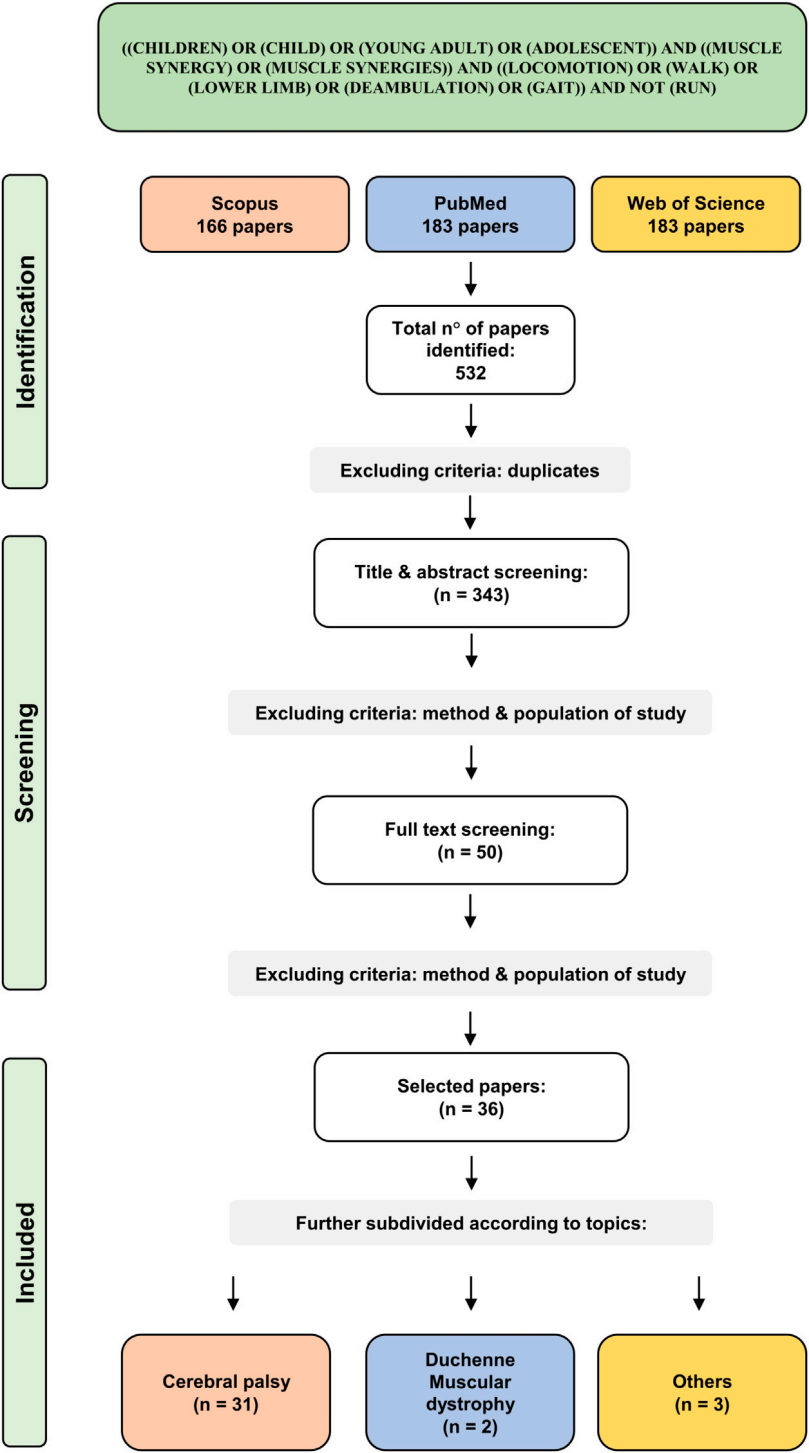
Prisma flowchart of the included studies.

As a result of the screening, 183 papers were found on PubMed, 166 on Scopus, 183 on Web of Science. The total number of articles was 532. After duplicate removal, 343 articles were screened. Title and abstract allowed to exclude 293 papers, mainly due to different target populations (i.e., age, diagnosis) or study design (i.e., no muscle synergy analysis, not involving lower limb, not analyzing locomotion). Full-text screening of the remaining 50 articles excluded 14 articles because of different target study purpose. In the end, 36 papers were included in the review. Selected articles were subdivided into three macro categories according to the pathologies analyzed: “Cerebral palsy”, “Duchenne muscular dystrophy” and “others”. The PRISMA chart for the study is reported in Figure 1.

**FIGURE 2:**
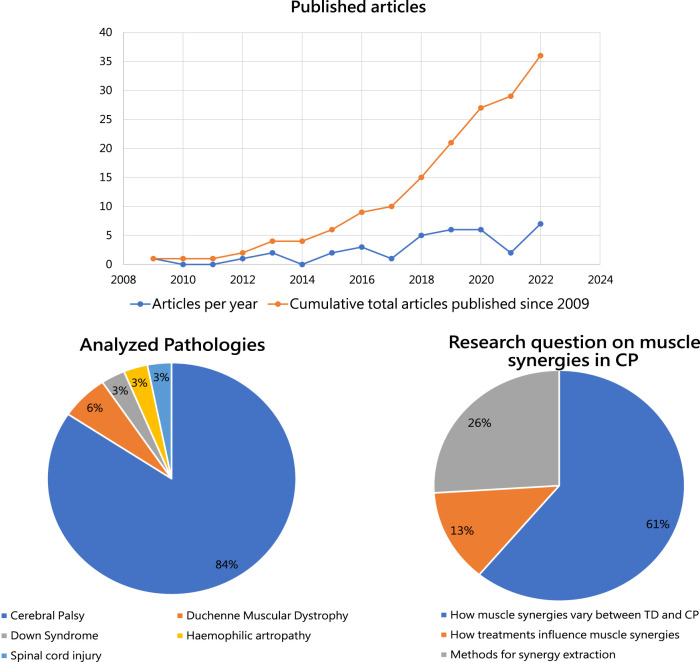
Upper panel: Number of studies on muscle synergies investigating children affected by developmental diseases per year, since 2009, is reported in blue. Cumulative number of articles published on muscle synergy analysis in children affected by DD eligible for this review is shown in orange. Lower left panel: The percentages of studies exploiting muscle synergy analysis for the considered developmental diseases. Lower right: The main research questions analyzed in the available articles on muscle synergies in CP.

Since 2018, a general increase in interest on muscle synergy analysis in children with disability was found. In 2019 and 2020, a total of 12 articles were published on the topic. The reduction of papers observed in 2021 is probably explainable with the COVID-19 pandemic rather than with a deflection of interest. In 2022, 7 articles were published on the topic, the highest number of eligible papers in a single year. Details on the number of published articles and on the cumulative number of articles are reported on the top of Figure 2.

The first article in our screening was published in 2009 and focuses on children with Down Syndrome. It aims at comparing the muscle activity patterns in toddlers with normal development and those affected by trisomy 21 (Chang et al., 2009), showing minimal differences between cases and controls. In the following years, literature has focused mainly on analyzing muscle synergies in CP: 31 out of the 36 articles included in the present review investigate the influences of CP on muscle synergies. Five articles investigate other DD to determine variation of muscle synergies from the ones observed in normally developed children (Figure 2, lower left panel).

The articles investigating muscle synergies in CP were further categorized according to their main research questions (Figure 2, lower right panel). The majority of the studies focus on differences between muscle synergies between TD and CP (a), agreeing in finding major differences in synergy composition at increased GMCSF level. 13% of the included studies focus on how treatment interferes with muscle synergies (b), demostrating important amelioration in gait biomechanics, but without clear changes in synergy structure. Lastly, 26% of the included articles focus on methods of synergy extraction (c), ranging from the role of EMG for muscle synergy analysis (Zwaan et al., 2012) to the recent work by d’Avella et al. posing questions on future directions for the field (D’Avella et al., 2022).

Reflecting our organization of the articles, Table 1 summarizes the articles that investigate how muscle synergies vary in children affected by CP and the methods of synergy extraction.

**TABLE 1:** Aim of the study, population, analyzed muscles, data processing, data structure, extracted synergies, main results of the articles investigating how muscle synergies vary in children affected by Cerebral Palsy (CP) in respect to healthy subjects. Acronyms: CP: cerebral palsy, TD: typically developed, HC: healthy controls, GMFCS: Gross Motor Function Classification System, BW: backward walking, FW: forward walking, SDR: selective dorsal rhizotomy, BoNT-A: Botulinum Toxin Type-A, GM: gluteus maximum; Gmed: Gluteus medius; BF: biceps femoris; ST: semitendinosus; SM: semimebranosus; RF: rectus femoris; VL: vastus lateralis; VM: vastus medialis; TA: tibialis anterior; PL: peroneus longus; EHL: extensor hallucis longus; SO: Soleus; ES: erector spinae; TFL; tensor fasciae latae; MG: medial gastrocnemius; LG: lateral gastrocnemius; GR: gracilis; RA: rectus abdominis, NMF: Non-negative matrix factorization, RMSE: Root mean square error, HP: high pass filter, LP: low pass filter, WNMF: weighted-NMF, Walk-DMC: dynamic motor control index during walking, VAF: Variance Accounted For.

Study	Aim of the study	Population	Analyzed muscles	Data processing				Extracted SYNERGIES		Data structure	Main results
				Processing	Threshold	Normalization	Algorithm	Healthy controls	Cerebral palsy		
Kim et al. (2022)	To examine if external environmental changes to the walking task influence muscle synergies in children with cerebral palsy (CP) and/or typical development (TD)	**CP:** n = 6 GMFCS I; n = 2	TA, EHL, LG, SO RF VL, ST. BF	HP = 35 Hz LP = 5 Hz	VAF >90%	Norm_window	NMF	**1:** TA + HL + RF + VL	**1:** TA + EHL + RF + VL + ST + SM	Concatenated_all	Muscle synergies in children with CP are more sensitive to changes in the external walking environment than in typically developing children
		GMFCS II; age 11–18yo						**2:** MG + SO + PL	**2:** MG + SO + PL + ndTA EHL + RF		
		**TD:** n = 8; age 11–18yo						**3:** ndTA + EHL + RF + VL	**3:** ndMG + SO + PL		
								**4:** ndMG + SO + PL	**4:** nd MG + SO + PL		
								No changes at walking environment changes	Alterations at treadmill walking speed variation		
Ettema et al. (2022)	To evaluate the effects of prolonged walking on signs of muscle fatigue and complexity of neuromuscular control in children with CP	**CP:** n = 10; GMFCS I – II; age 6–18yo; no surgery within 6 months **TD:** n = 15; age 6–18yo	RF, VL, ST, GM, LG, PL, TA and SO	HP = 20Hz; LP = 10 Hz	VAF1 Synergies	Norm_all	NMF	**1:** LG + MG + SOL + PL	**1:** starts earlier	Single_stride	Children with CP presented signs of muscle fatigue after prolonged walking, while no effects were found for TD. Still muscle fatigue is not associated to changes in muscle synergies’ complexity
								**2:** ST + SM	**2:** almost the same		
								**3:** VL + RF	**3:** the 2nd peak is delayed		
								**4:** TA + GL + SOL + PL	**4:** the 2nd peak is delayed and prolonged in time		
									Same weighting, different timings		
Spomer et al. (2022)	To evaluate the effect that altered biomechanics have on synergies. (*Emulated equinus EE; CP equinus CP-Q; emulated equinus crouch EEC; CP equinus crouch CP-EEC; emulated mid crouch and crouch EmidC; CP mid crouch and crouch CP-EmidC*)	2 **CP** subjects matched to every healthy subjects for emulated gait **HC:** n = 14; mean age: 23yo	GM, BF, ST, SM, VM, SO, TA and MG	HP = 20Hz; LP = 10 Hz	VAF1 VAFn	Normalized to the 95th percentile of maximum activation	NMF	**1:** Glu + VL + RF	***EE:*** 1: TA; 2: BF + ST + SM; 3: GL + GM	Single_stride	Altered gait patterns are not primarily driving the changes in synergies observed in CP
								**2:** ST + SM + BF	***CP -E***: TA; 2: ST + GM + LG + VL		
								**3:** SOL + MG + LG	***EEC:*** 1: TA; 2: BF + ST; 3–4: TA + GL + GM + VL		
									***CP-EC:*** 1.: TA; 2.: ST + SM + VAL + GM + GL		
									***EmidC:*** 1.: TA; 2.: ST + LG + MG + TA; 3.: GM + GL + TA; 4.: VL		
									***CP-EmidC:*** 1.: TA; 2.: ST + SM + VL + GM + GL		
Goudriaan et al. (2022)	To determine if muscle synergy structure differs between gait patterns	**CP**: n = 188; age 3–17yo **TD**: n = 57; age 4–17yo	RF, VL, BF, ST, SM, TA, MG, LG, SO and GMed	HP = 20 Hz LP = 10 Hz	VAF>90% (and others) VAF1	Normalized to its average amplitude	WNMF	n/a	In all pathways: **Stance phase:** VL + BF + ST + SM + Glu **Push off**: MG + SO + Glu **Swing phase:** RF + TA	Concatenated_all	Synergy structure was similar between gait patterns, although weights differed in the more impaired children (crouch and jump gait) when compared to the other patterns
Kim et al. (2021)	To conduct a larger study incorporating more muscles and gait cycles to prove that children with CP have greater stride-to-stride variability	**CP**: n = 10; age 14.9 ± 3.8 yo; GMFCS I – II; no surgery within 6 months **TD**: n = 10; age 15.0 ± 3.2 yo	TA, EHL, LG, SO, RF, VL, ST and BF	HP = 35 Hz LP = 6 Hz	VAF>90%	Norm_all	NMF	T1: TA + EHL T2: SO + PL + MG T3: nd HL + TA + ST + RF + VL T4: RF + VL + TA + EHL + ST T5: nd HL + TA + ST + RF + VL T6: MG + SO + PL + CP 1 – 7 – 10 + N: MG + SO + PL	7 CP specific clusters + T1 – 3 – 4 – 5 – 6 + N: MG + SO + PL	Concatenated_all	A greater CP-specificity of muscle synergies was related to poorer performance. CP-specific synergies can influence motor dysfunction
Bekius et al. (2021)	How muscle synergies differ in children with CP vs. TD children with the same walking ability, before and after the onset of independent walking	**CP:** n = 20; age 6.5–45.5months **TD:** n = 20; age 6.3–53.5 months	TA, GM, LG, SO, RF, VM, VL, BF, TFL, MG, ES	HP = 30 Hz LP = 10 Hz	VAF>85% or +8% + 80% single muscle	Maximum of mean value	NMF	**Supported walking:** 3–4 synergies **Indipendent walking:** 3–4-5 synergies	**Supported walking:** 2–3-4 synergies **Indipendent walking:** 3–4 synergies	averaged	Early brain lesions result in early alterations of neuromuscular control, specific for the most affected side in asymmetric CP
Cappellini et al. (2020)	To consider how to intervene according to how much the dysfunction of gait can be related to spinal neuronal networks vs supraspinal dysfunction	n/a	n/a	n/a	n/a	n/a	n/a	n/a	n/a	n/a	Physical therapy interventions, recent advances in biotechnology and neuromodulation of the locomotor circuitry might promote early motor recovery in children with CP
Short et al. (2020)	To evaluate the relationships between cortical and muscle activation	**CP:** n = 9; age 16.0 ± 2.7 yo; GMFCS I – II **TD:** n = 12; age 4.8 ± 3.0 yo	TA, MG, SO, PL, RF, VL, ST, SM and EHL	HP = 35 Hz LP = 5 Hz	VAF>90%	Normalized single stride	NMF	**1:** TA + EHL + RF + VL + ST	Same 5 synergies as TD children, but synergy n.3 appears later, while the 5th one is anticipated	Averaged_all	Differences reflecting the unilateral injury that primarily disrupts distal control and its cortical representation in the sensorimotor brain regions in CP
								**2:** RF + VL + ST + SM			
								**3:** MG + SO + PL			
								**4:** RF			
								**5**: ST + SM + EHL (action of MG of the non-dominant leg)			
Steele et al. (2019)	To prove if EMG recordings and muscle synergies are repeatable between visits	**CP:** 20 children with bilateral CP	RF, ST, SM, VL, TM, MG	HP = 25 Hz LP = 10 Hz	VAF>95%	Norm all	NMF	n/a	n/a	Averaged all	The inter-visit variance ratios of EMG data for children with CP were similar to previously reported results for typically developing children and unimpaired adults
Yu et al. (2019)	To assess the relationship between GMFCS levels and the gait synergistic control	**CP:** n = 18, age 4–6 yo; GMFCS I – II – III; No suergery within 1 year **TD:** n = 8; age 4.43 ± 1.36 years	TA, SO, LG, VL, RF, ST, BF and TFL	HP = 50 Hz LP = 10 Hz	Forced to four	Norm_all	NMF	4 synergies per leg	GMFCS I – II: Same 4 synergies as described in TD children, different pattern of activity: Synergies n1 n2 last longer, without evident peaks Synergy n3 does not show the first peak GMFCS III: Merging synergies/new synergies	Single_stride	Children at GMFCS levels I and II and the TD children had similar synergy structures, with different synergy activations. Children GMFCS level III could not access all four basic synergies on both sides
								**1:** VL + RF + TFL			
								**2:** SO + LG			
								**3:** TA			
								**4:** ST + SM + BF			
Booth et al. (2019)	To establish if muscle synergies are modified when children with CP are challenged to improve aspects of gait with real-time biofeedback	**CP:** n = 25; age 5–16yo; GMFCS I II; no surgery within 1year **TD:** n = 27; age 5–16yo	GMed, RF, VL, ST, TA, MG, SO and PL	HP = 20 Hz LP = 10 Hz	VAF>90%	Mean muscle activation	NMF	4 synergies solution for VAF >90% in 13 children 1 child with 5 synergies	3 synergies solution for VAF >90% in 13 children; the others: 2–3 synergies No significant interaction effect of feedback on composition muscle weightings and timings vs*.* kinetic and kinematic parameters	Concatenated_all	Children with CP can selectively adapt and improve aspects of gait when challenged with biofeedback
Cappellini et al. (2018)	To evaluate muscle synergies in hemiplegic and diplegic children vs. typically developed peers during forward walking (FW) and backward walking (BW)	**CP:** n = 14; age 3–11.1 yo; GMFCS I – II – III; No surgery within 1year **TD**: n = 14; age 3.3 – 11.8yo	LG, MG, SO, TA, BF, ST, TFL, GM, RF, VL and VM	HP = 30 Hz LP = 10 Hz	VAF linear fit (RMSE<10^–4^)	Norm_all	NMF	**FW:** SOL + LG + MG: late stance **BW:** BF + ST: mid swing	**FW:** SOL + LG + MG at whole stance. Different CoA: plantarflexors and TA between legs **BW: d**ifferent CoA: RF, VL, VM, Glu, BF, ST, TA Different CoA: ST + SM between legs	Concatenated_all	BW highlights prominent gait asymmetries in children with CP, giving a more comprehensive assessment of the gait pathology No relevant gait asymmetries have been found during FW.
Hashiguchi et al. (2018)	To compare the number of muscle synergies between children with CP and TD children and clarified whether certain clinical parameters differed accordingly	**CP:** n = 13; GMFCS I – II – III **TD:** n = 10; age: 6 – 18yo	TA, LG, SO, Gmed, RF, VM, BF and ST	HP = 20 Hz LP = 10 Hz	VAF >90%	Norm_all	NMF	4–5 synergies	2–3-4 synergies	Concatenated_all	Children with cerebral palsy had significantly fewer synergies than children developing typically. The extent of spasticity and gait kinetics differed according to the number of synergies
Cappellini et al. (2016)	To evaluate spatiotemporal alpha-motoneuron activation during walking by mapping the electromyographic activity profiles from several, simultaneously recorded muscles	**CP**: n = 35; age 2 – 12yo; GMFCS I – II – III; no surgery within 1year **TD:** n = 7; age1 – 1.2 yo; n = 26 age 1–2 – 12yo	BF, GM, LG, MG, RF, SO, VL, ST, TA, TFL and VM	HP = 30 Hz LP = 10 Hz	VAF linear fit (RMSE<10^–4^)	Norm_all	NMF	SO + LG + MG: late stance TA: 2 peaks in ST + BF	SO + LG + MG ++: whole stance TA: 1 peak at early swing RF + VL + VM ++ (vs. ST BF: shifted later) Wider EMG bursts: ST, SM, BF, VL, SO, LG, MG	Concatenated_all	Early injuries to developing motor regions of the brain substantially affect the maturation of the spinal locomotor output and consequently the future locomotor behavior
Shuman et al. (2016)	To evaluate the repeatability of synergy complexity and structure in unimpaired individuals and individuals with cerebral palsy (CP)	**CP:** n = 5; age 10.2 ± 2.3 **TD:** n = 5, age 10.3 ± 3.5	GMed, ST, SM, BF, VL, RF, GL, SO, TA	HP = 40 Hz LP = 4 Hz	VAF 1	Norm_all	NMF	n/a	n/a	Single_stride	Synergies are repeatable between days in both groups
Tang et al. (2015)	To explore the mechanism of lower extremity dysfunction of CP children through muscle synergy analysis	**CP:** n = 12; age 5.75 ± 1.83 yo; no history of surgery **TD:** n = 8; age 6.05 ± 1.66yo **HC:** n = 10; age 24.5 ± 1.08 yo	TA, SO, LG, VL, RF, ST, BF and TFL	HP = 50 Hz LP = 10 Hz	VAF >95%	Norm to unit variance	NMF	**HC:** 4 synergies per leg	The higher the GMCSF level, the lower the number of “classical” synergies exploited, which are replaced by CP-related synergies. In toe-walkers: SO + LG + TA	Single_stride	Fewer mature synergies were recruited in the CP group and many abnormal synergies specific to the CP group appeared with a larger difference in structure and symmetry between two legs of one subject and different subjects
								1: VL + RF + TFL			
								2: ST + SM + BF			
								3: SO + LG			
								4: TA			
								**TD:** 3 synergies, asymmetrically recruited between legs			
Steele et al. (2015)	To evaluate if individuals with CP demonstrate reduced complexity of neuromuscular control during gait and if changes are related to functional ability	**CP:** n = 549; no previous surgery **TD:** n = 84; age: 3.9 – 70yo	RF, ST, SM, BF, MG and TA	HP = 20 Hz LP = 10 Hz	VAF1, walk-DMC	Norm single stride	NMF	3 synergies equals a VAF >90% for 60% of individuals	1–2 synergies equals a VAF >90% in 80% of CP patients: 1: ST + SM + MG (++ in early stance) 2: RF + TA (-- in late swing)	Single_stride	Individuals with CP use a simplified control strategy during gait compared with unimpaired individuals, similarly, to stroke survivors
								1: BF + ST + SM			
								2: MG			
								3: RF + TA			
Li et al. (2013)	To find the difference of lower-limb muscle synergies between CP children and adults	**CP:** n = 8; no previous surgery **HC:** n = 5	TA, SO, LG, VL, RF, ST, BF and TFL	n/a	VAF	Norm sub-maximal	NMF	All subjects exploit 5 “classical” synergies per leg	A combination of 3 “classical” synergies per leg + specific CP synergies	n/a	Four or five muscle synergies were required for adult subject’s vs. two, three or four for cerebral palsy
								1: VL + RF + TFL	1: TA + SOL + LG		
								2: ST + SM + BF	2: VL + RF + TSF + BF + ST		
								3: SO + LG			
								4: TA			
								5: VL + RF			
Zwaan et al. (2012)	To evaluate the role of EMG in studying the thigh and the extensor synergies	**CP:** n = 39; age 2–13yo pre and post SDR n = 38; age 3–20yo (GMFCS I-II-III) **TD:** n = 30; age 6–11yo	VM, ST and MG	HP = 20 Hz LP = 2 Hz	n/a	n/a	Cross correlation	**Thigh synergy**: VM vs. ST **Extensor synergy:** GM vs. VM	The extensor synergy is more expressed	Averaged_all	The EMG is sensitive enough to represent an aberrant motor control in CP
Sorek et al. (2022)	To evaluate the influence of the number of muscles and strides on estimating motor control accuracy during treadmill-gait, in individuals with cerebral palsy (CP)	**CP:** 44 children and adolescents	GMed, ST, RF, VL, MG, SO, TA, PL	HP = 20 Hz LP = 10 Hz	VAF1, VAF >90%	Norm_to_mean amplitude	WNMF	n/a	n/a	Concatenated_all	Differing numbers of muscles and strides did not influence the group mean tVAF1 value, but it influenced the tVAF-threshold value. Using different number of muscles or strides can lead to a large measurement error in the individual tVAF1 value
Rabbi et al. (2022)	To estimate muscle synergies from EMG measured from a small set of muscles	**CP:** 6 children**TD:** 6 children	LG, MG, SO, TA, RF, SM, BF, VL, VM	HP = 30 Hz LP = 6 Hz	VAFn, *R* ^2^, RMSE, Kolmogorov-Smirnov test	Norm_all	NMF	n/a	n/a	Concatenated_all	Muscle activation patterns of unmeasured muscles can be estimated from EMG measured from three to four muscles using muscle synergy extrapolation method
d'Avella et al. (2022)	A commentary on “Muscle structure and gait patterns in children with spastic cerebral palsy”	n/a	n/a	n/a	n/a	n/a	n/a	n/a	n/a	n/a	Spatiotemporal decomposition of combined kinematic and EMG data and extracting task-specific features of motor variability affecting performance might provide compact and discriminative representations of individual motor control strategies
Falisse et al. (2020)	To evaluate the ability of a predictive simulation platform to differentiate the effects of musculoskeletal and motor control impairments on the impaired walking pattern	one CP subject aged 10-15y	GMed, RF, BF, ST, TA, GL, VL, SO	n/a	n/a	Personalized muscle-tendon parameters	n/a	n/a	n/a	n/a	The altered muscle-tendon properties rather than reduced neuromuscular control complexity and spasticity were the primary cause of the crouch gait pattern
Kim et al. (2018)	To investigate the variability of muscle synergy in children with CP vs. TD, by extracting them from individual strides and allowing the n. of synergies to vary for each stride. Clustering and discriminant analyses were then applied	**CP:** n = 20; age 12.5 ± 3.3 yo; GMFCS I – II; no surgery within 1year **TD:** n = 8; age 12.0 ± 2.6 yo	TA, MG, RF, ST and SM	HP = 35 Hz LP = 5 Hz	VAF >90% (and other); VAF1; walk-DMC	Normalized single stride	NMF	4 clusters of synergies to describe 5 strides	10 clusters of synergies to describe 5 strides. One cluster is CP specific: TA + RF of non-dominant leg TA + RF + ST + SM of dominant leg During stance (0%–40% of the gait cycle)	Single_stride	Children with CP utilize the same synergies as those with TD in some strides while at other times exhibiting distinct synergies do not present in those with TD
Shuman et al. (2017)	To evaluate how EMG signal processing impacts synergy outputs during gait, by evaluating the impacts of two common processing steps for synergy analyses: low pass (LP) filtering and unit variance scaling	**CP:** n = 40 (GMFCS I), n = 40 (GMFCS II = n = 33 (GMFCS III); age 10.9 ± 5.8 yo **TD:** n = 73; age 10.5 ± 3.5yo	RF, ST, BF, MG, TA	HP = 40 Hz LP = 4-6-8-10–20-30–40 Hz	VAF >90%; tVAF; Z-score of tVAF	Norm_all; unit variance	NMF	TA + RF	TA + MG	Concatenated_all	Unit variance scaling caused comparatively small changes in tVAF. Synergy weights and activations were impacted less than tVAF by LP filter choice and unit variance normalization
								ST + BF	TA + RF		
									ST + BF		
van der Krogt et al. (2016)	To determine the effect of different choices in the EMG analysis (filtering, normalization, and stride selection) on the outcome of synergy analysis the effects on synergies before and after botulinum neurotoxin (BoNT-A) treatment	68 ambulant children with CP (9.3yo) Pre and post treatment with botulinum toxin - A	RF, VL, ST. TA, MG	HP = 20 Hz LP = 2-3-10–25 Hz	VAF; VAF1	Norm_all_not_ normalized; Norm_pre_treatment	NMF	n/a	VAF decreased strongly with increasing filter frequency, and when including individual strides rather than the mean. No main effect of normalization was found. VAF increased slightly after BoNT-A	Single stride; averaged_all; concatenated_all	Synergy outcomes strongly depend on EMG processing method, and may help in making deliberate choices during data analysis

* analyzed muscles are not reported because no clear information about the extracted synergies have been indicated.

Table 2 focuses on how longitudinal treatments influence muscle synergies in children affected by CP. Table 3 presents the articles showing how muscle synergies vary in other developmental diseases, and the methods of synergy extraction.

**TABLE 2:** Aim of the study, Population, Analyzed muscles, Data processing, Data structure and main results of the articles investigating how muscle synergies vary in children affected by CP after treatments. Acronyms: SDR: selective dorsal rhizotomy, BTA Botulinum Toxin Type-A Injection, SEMLS: single-event multilevel orthopedic surgery; CONS: conservative treatment, ORTHO: single-level orthopedic surgery, CP: cerebral palsy, GMFCS: Gross Motor Function Classification System, HP: high pass filter, LP: low pass filter, VAF: Variance Accounted For, Walk-DMC: dynamic motor control index during walking, NMF: Non-negative matrix factorization, WNMF: weighted-NMF, Gmed: Gluteus medius; BF: biceps femoris; ST: semitendinosus; SM: semimembranosus; RF: rectus femoris; VL: vastus lateralis; VM: vastus medialis; TA: tibialis anterior; G: gastrocnemius; S: soleus; MG: medial gastrocnemius.

Study	Aim	Population	Analyzed muscles	Data processing				Data structure	Main results
				Processing	Threshold	Normalization	Algorithm		
Pitto et al. (2020)	To predict and compare the functional outcome of a series of candidate interventions	**BTA**: n = 25; age 8.3 ± 2.1yo	RF, VL, BF, TA, Gmed, SM, G, S, ST	HP = 40Hz; LP = 6 Hz	VAF_based bootstrap procedure	To have unitary standard deviation	NMF	Concatenated_all	Subject-specific muscle synergies computed from pre-treatment EMG data could be used with confidence to represent the post-treatment motor control of children with CP during walking
		**SEMLS:** n = 21; age 11.5 ± 3.1yo							
Shuman et al. (2019)	To understand if synergies change after treatment, or are associated with treatment outcomes	**BTA**: n = 52, age 6y10 m (±2y11 m)	Gmed, RF, VL, SM, ST, TA, G, S, BF, ST	HP = 20Hz; LP = 10 Hz	VAF >90%; VAF1; walk-DMC	Norm_all	WNMF	Concatenated_all	There were minimal changes in synergies after treatment despite changes in walking patterns
		**SDR**: n = 38, age 9y4m (±2 years)							
		**SELMS**: n = 57, age 12y2m (±3y1m)							
Oudenhoven et al. (2019)	To identify factors associated with long- term improvement after selective dorsal rhizotomy	**SDR:** n = 36	RF, VL, ST, TA, MG	HP = 20Hz; LP = 2 Hz	VAF >90%	Norm_single stride	NMF	Concatenated_all	Gait quality improved after SDR, with a large variation between patients. Gait improved more in children with GMFCS I and II compared to III
Shuman et al. (2018)	To determine whether patient-specific differences in muscle synergies were associated with changes in gait after treatment	Center 1	RF, SM, G, TA Four additional muscles: Gmed, VL, ST, S	HP = 20 Hz LP = 10 Hz	VAF1; walk-DMC	Norm_all	WNMF-NMF	Concatenated_all	Children with less impaired motor control were more likely to have improvements in walking speed and gait kinematics after treatment, independent of treatment group
		**CONS:** n = 76, age 6y8m (±2 years,7 m)							
		**ORTHO:** n = 39, age 6y11 m (±3y4m)							
		**SEMLS:** n = 176, age 10y0m (±3y5m)							
		**SDR**: n = 182, age 5y7m (±2y0m)							
		Center 2							
		**BTA**: n = 60, 6y9m (±2y11 m)							
		**SEMLS:** n = 59, 12y1m (±3y1m)							
		**SDR:** n = 44, 9y1m (±2y0m)							

**TABLE 3:** Aim of the study, population, analyzed muscles, data processing, data structure, extracted synergies, main results of the articles investigating how muscle synergies vary in children affected by other developmental diseases in respect to healthy controls. Acronyms: DMD: Duchenne muscular dystrophy, CP: cerebral palsy, PWHA: people with hemophilic arthropathy, ISCI: Incomplete Spinal Cord Injury, ID: intellectual disability, DS: Down Syndrome, TD: typical development, GMFCS: Gross Motor Function Classification System, GM: gluteus maximum; Gmed: Gluteus medius; BF: biceps femoris; ST: semitendinosus; SM: semimebranosus; RF: rectus femoris; VL: vastus lateralis; VM: vastus medialis; TA: tibialis anterior; SO: Soleus; ES: erector spinae; MG: medial gastrocnemius; LG: lateral gastrocnemius; RA: rectus abdominis, NMF: Non-negative matrix factorization, RMSE: Root mean square error, HP: high pass filter, LP: low pass filter, WNMF: weighted-NMF, Walk-DMC: dynamic motor control index during walking, VAF: Variance Accounted For.

Study	Aim	Population	Analyzed muscles	Data processing				Data structure	Extracted SYNERGIES		Main results
				Processing	Threshold	Normalization	Algorithm		TD	DD	
Vandekerckhove et al. (2020)	To determine if synergies are altered in Duchenne muscular dystrophy and if these alterations could be linked to muscle weakness	**DMD:** n = 22; age 6–10yo; non-previous limb surgery	GMed, RF, ST, SM, TA and MG	HP = 20 Hz LP = 10 Hz	VAF >90% (and others); VAF1	Normalized to its averaged amplitude	NMF	Single_step	Early stance and late swing: Glu + ST	Increased activity of RF	Synergy weights and activations do not change in DMD
		**TD:** n = 22; age 7–10yo	SO, VL, BF						Mid stance: MG + LG	Decreased activity of LG and MG	
									End stance and swing: TA + RF		
Goudriaan et al. (2018)	To compare the impact of muscle weakness on muscle synergies	**CP:** n = 15; mean age 8.9yo; GMFCS I-II; no previous surgery	RF, VL, ST, SM, BF, MG, SO, TA and Gmed	HP = 20 Hz	VAF1	Normalized to its averaged amplitude	NMF	Concatenated_all	Mean tVAF1: 0.65	Mean tVAF1 in DMD: 0.65	Non-neural muscle weakness has little influence on complexity of motor control during gait
		**DMD:** n = 15; mean age 8.7yo		LP = 10 Hz						Mean tVAF1 in CP: 0.60	
		**TD**: n = 15; mean age 8.6yo									
Cruz-Montecinos et al. (2019)	To assess how haemophilic arthropathy affects the complexity of neuromuscular control during gait and their relation	**PWHA:** n = 13; age 18–45yo	MG, LG, SO, TA, VL, VM, RF, ST, BF, GM and Gmed	HP = 20 Hz LP = 10 Hz	VAF >90% or <+5%; walk-DMC	Norm_all	NMF	Concatenated_all	Acceptance synergy: VL + VM + RF; ST + BF	Acceptance synergy: VL + VM + (less) RF; SM + BF (more)	PWHA reduces the complexity of neuromuscular control. The higher the number of joints involved, the more limited the synergies are
		**TD:** n = 13; age 18 – 45yo							Push off synergy: MG + LG + SOL	Push off synergy: MG + LG + SOL; VL + ST	While considering 1 synergy in PWHA it can describe a higher tVAF value
									Swing synergy: TA + RF	Swing synergy: TA + RF	
									Deceleration synergy: TA and ST + SM + BF	Deceleration synergy: TA and ST + SM + BF	
Fox et al. (2013)	To examine neuromuscular control of reciprocal locomotor tasks in children with ISCIs as well as children without neurological injuries	**ISCI:** n = 5; age 3–13yo	TA, MG, VM, RF, ST, SM and GM	HP = 30 Hz LP = 4 Hz	VAF >90%	Normalized to that muscle’s mean value during that trial	NMF	Single stride	Early stance: VM + Glu	Stance: MG + TA	Children with ISCIs most often required two modules and relied on synergistic muscle coactivation across the flexion and extension phases of each task
		**TD:** n = 5; age matched							Late stance: MG	Swing: ST, SM	
									Early swing and late swing-stance: TA + RF		
									Swing and early stance: ST, SM		
Chang et al. (2009)	To compare the emergence of muscle activity patterns of toddlers with Typically Development (TD) to those of toddlers with Down Syndrome (DS)	**DS:** n = 8	TA, MG, LG, RF, BF, RA, ES	n/a	n/a	n/a	n/a	n/a			Both TD and DS groups similarly demonstrated the need for a prolonged period of practice (6 months) to acquire a rhythmic and stable muscle activation pattern during independent walking
		**TD:** n = 8									By 6 months, TD show an efficient synergy among muscles, allowing increased relaxation time between bursts. Toddlers with DS improved the rhythmicity of their muscle burst, sustaining longer bursts but timing remained inconsistent

### 3.2 What is known on muscle synergies in developmental diseases

#### 3.2.1 Cerebral palsy

Since the first pilot studies that investigated muscles synergies in DD, it was clear that the kinetic and the kinematic variables observed in children affected by CP were different from those observed in typically developing children and this was reflected into muscle synergy patterns. In fact, children affected by CP use simplified control strategies during gait, exploiting fewer muscle synergies compared to normally developing children (Li et al., 2013; Steele et al., 2015; Tang et al., 2015; Hashiguchi et al., 2018). In addition, children affected by CP use some synergies exploited by healthy peers as well as specific synergies which are unique and repeatable over multiple gait cycles and between days **(** Shuman et al., 2016; Kim et al., 2018; Steele et al., 2019).

Two or three muscles synergies are sufficient to describe locomotion in CP. The observed motor modules are either similar to the healthy ones, or they are the result of merging synergies, giving rise to “CP-specific” motor modules. On the contrary, some modules are often retained: the most repeatable synergies across different CP subjects are the ones with high loadings for the triceps surae in the late stance phase and the one showing the coactivation of the tibialis anterior-rectus femoris with the hamstrings, as it is reported in the previous tables.

According to literature (Table 1), gait kinematic and muscles fatigability are not primary driving changes for muscle synergy composition, while the severity of the disease (represented as a high GMFCS level) negatively interferes with the number and composition of the exploited synergies. Indeed, according to many studies, the differences in muscle synergies observed in TD children and children affected by CP are not related to the fatiguability of the muscular tissue nor to impaired biomechanics (Yu et al., 2019; Falisse et al., 2020; Ettema et al., 2022; Goudriaan et al., 2022; Spomer et al., 2022). To support the hypothesis, it is noteworthy to consider that different treatment approaches have shown minimal changes in synergies, even if gait pattern, movement quality and speed improved significantly after treatment (Table 2). These results are coherent with the fact that CP results from a cortical and/or subcortical brain lesions in the perinatal period, causing impairments in motor controls since the very beginning (Cappellini et al., 2016; Cappellini et al., 2020; Short et al., 2020; Bekius et al., 2021).

#### 3.2.2 Other developmental diseases

Two studies focus on Duchenne muscular dystrophy (DMD), demonstrating no differences in the recruitment of muscles synergies (Goudriaan et al., 2018; Vandekerckhove et al., 2020) between cases and controls. Cruz-Montecinos and others studied how muscle synergies change in children affected by hemophilic arthropathy, showing that there is a direct correlation between the number of affected joints and the impaired neuromuscular control (Cruz-Montecinos et al., 2019). Chang et al. demonstrated that toddlers affected by Down syndrome do not correctly adapt to external changes (Chang et al., 2009) whereas Fox et al. showed that children affected by incomplete spinal cord injury used fewer muscles synergies compared to healthy peers (Fox et al., 2013). More details are reported in Table 3.

### 3.3 Analyzed muscles

**FIGURE 3:**
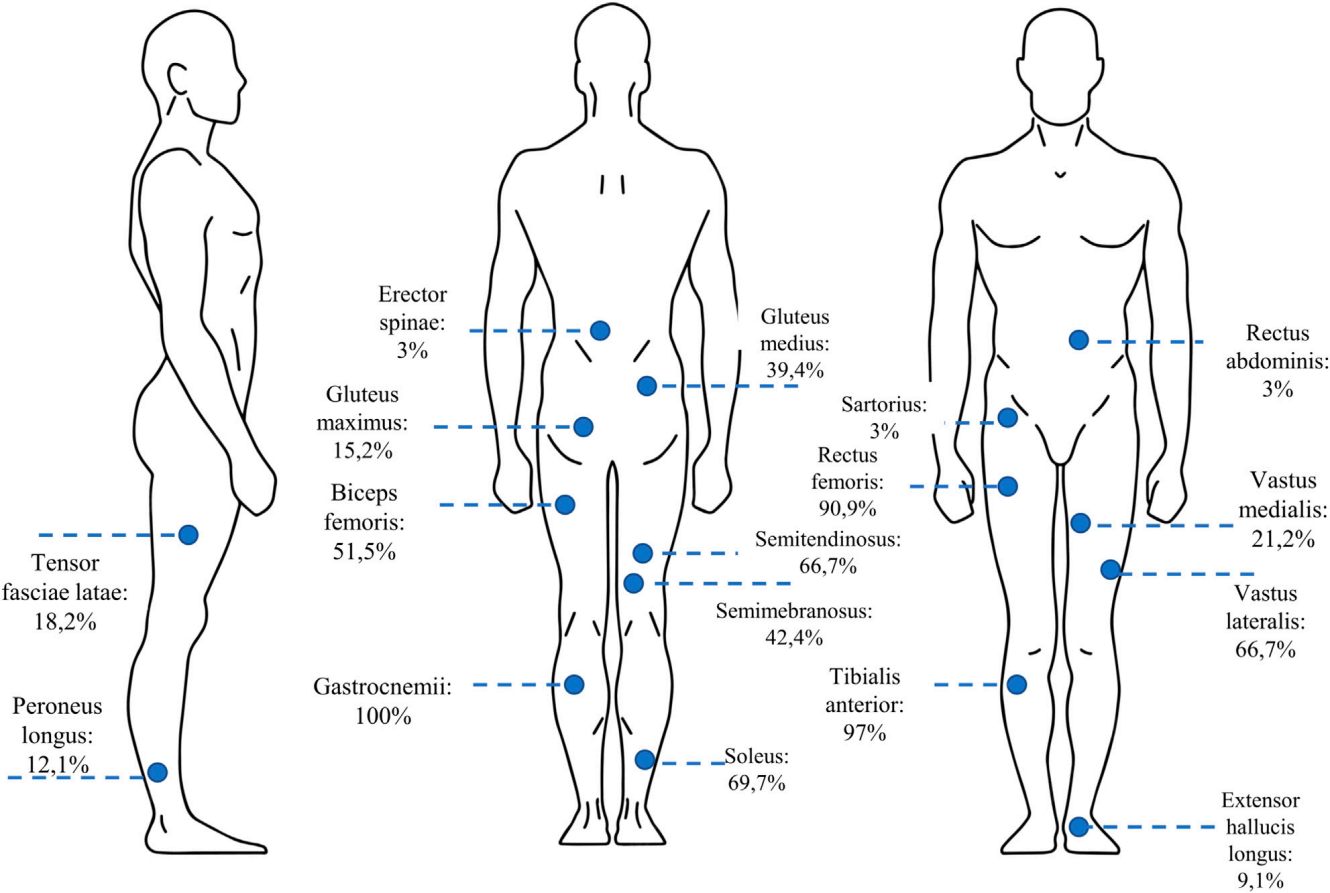
The percentage of the studies in which each of the lower-limb muscles was selected for muscle synergy analysis.

One of the important methodological aspects that must be considered in understanding muscle synergy variation is the number and type of analyzed muscles, as remarked by Steele and others (Steele et al., 2013) and, more recently, by Sorek and others (Sorek et al., 2022), that show how the selection and the number of selected muscles may impact on the extracted synergies. The studies included in the present review analyzed mainly the following muscles: gastrocnemii (100%), the soleus (69,7%), the rectus femoris (90,9%), the biceps femoris (69,7%), the semitendinosus (90,9%), the gluteus maximus (15,2%) and medius (39,4%), the tibialis anterior (97%). Figure 3 shows a detailed chart of the muscles selected in the screened studies.

The selection of the muscles is coherent with what observed in reference studies of the field such as those by Ivanenko and others, who described human locomotion as a subsequent activation of 4–5 muscle synergies (extracted with Principal Component Analysis—PCA), that include the previously mentioned muscles (Ivanenko et al., 2004), and Clark and others (Clark et al., 2010), that described healthy locomotion with four to five synergies when using Non-negative Matrix Factorization (NMF) for extracting muscle synergies.

### 3.4 Data processing

In Tables 1–3, the details for data processing for all the studies included in the screening are reported.

#### 3.4.1 EMG processing

EMG processing includes the steps needed to obtain EMG envelopes before synergies are extracted. The first step is signal filtering. In our screening, we noticed that 20 Hz is the most used frequency for high-pass filtering (in agreement with EMG filtering recommendations, de Luca et al., 2010), while 10 Hz is frequently adopted for low-pass filtering as a typical cut-off frequency for achieving envelopes in muscle synergy analysis. However, even lower frequencies are used in many studies; it is known that EMG pre-processing may impact on muscle synergy outcomes. In fact, the high pass filtering reduces motion artefacts, but may slightly impact also on useful EMG signal; the low pass filtering influences the smoothness and regularity of EMG envelopes and may impact synergy number and composition.

In their work, Hug and others (Hug et al., 2012) observed that a wide variety of low-pass filters have been used in literature aiming at extracting muscle synergies during walking and they observed that the smoothing procedure influences the amount of VAF explained, and, as a consequence, the number of extracted synergies. They suggested caution when comparing the number of synergies from studies that adopt different filtering. They also suggested that when different velocities are compared, the low-pass cut-off frequency should be adapted to provide the same smoothing EMG profiles, even if this approach was rarely adopted.

In their paper, Shuman and others investigated low pass (LP) filtering and unit variance scaling influence synergy extraction (Shuman et al., 2017), by applying LP filters to the EMG data with cutoff frequencies ranging from 4 to 40 Hz. They showed that the total variance accounted for (tVAF) by a given number of synergies was sensitive to LP filter choice and decreased in both TD and CP groups with increasing LP cutoff frequency. They also concluded that the change in tVAF derived from filtering can alter the number of synergies selected for further analyses. Synergy weights and activations were impacted less than tVAF by LP filter choice. These results demonstrate that EMG signal processing methods impact outputs of synergy analysis and z-score based measures can assist in reporting and comparing results across studies and clinical centers.

The issue was investigated also by another work (van der Krogt et al., 2016), that studied what is the effect of different choices in the EMG filtering, on the outcome of synergy analysis in CP, by comparing low-pass filtering at 2, 3, 10, or 25 Hz. They found that the VAF decreased significantly with increasing filter frequency impacting on the number of extracted synergies.

#### 3.4.2 EMG normalization

**FIGURE 4:**
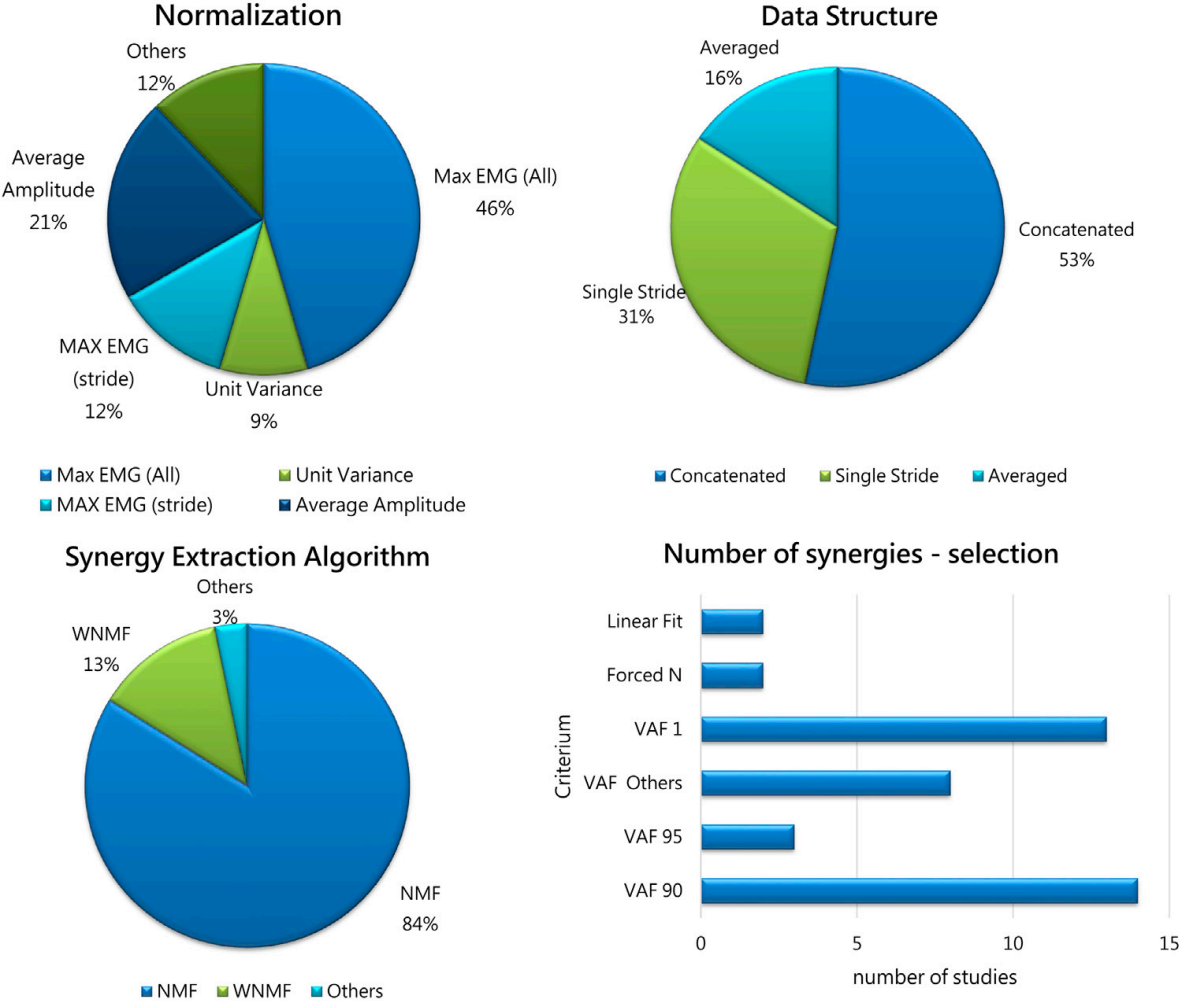
Upper-left panel: The percentage of Normalization Approaches adopted in the selected studies. Max EMG (All): normalization on the Maximum pre-processed EMG value on all trials; Average Amplitude: normalization on the average value for each channel; Unit Variance: normalization to achieve unit variance for each channel; Max EMG (Stride): normalization on single gait cycles; Others includes approaches used in single studies (maximum of mean value, normalized to the 95th percentile of maximum activation). Upper-left panel: Data Structure occurrences before synergy extraction: Concatenated (53%); Single Stride (31%); Averaged strides (16%). Lower-left panel: The percentage of Algorithms used in the selected studies. Acronyms: NMF: non-negative matrix factorization; WNM: weighted non-negative matrix factorization; Others: EMG cross correlation. Lower-right panel: The percentage of criteria for selecting the number of synergies used in previous studies: VAF threshold was adopted by most of the studies (almost 90%); 2 studies used the linear fit, and 2 forced *a priori* the number of extracted synergies.

Normalization of EMG envelopes is needed to allow inter-subject and inter-session comparisons, and to account for muscles that intrinsically produce less activity due to their properties. Normalization analyses included several approaches that are summarized in Figure 4 (upper-left panel). The most frequent approach is to normalize with respect to the maximum EMG value found in all the dataset for each muscle (46%). This approach was already in use for upper-limb studies (d'Avella et al., 2003), and is particularly effective when a reasonable high number of strides or walking conditions are available. Other approaches aim at normalizing EMG signals by the average value found for that muscle in all the trials (21%), to refer normalization to single strides rather than global approaches (12%) or to achieve unit variance for each channel (9%). Other approaches were also used (12%), and include normalizing on the 95% of mean of the peaks for each channel. No study employed the Maximum Voluntary Contraction (MVC) as it is hardly measured, especially on patients.

According to the available studies, non-uniformity in normalizing methods is however tolerable as it was reported that no main effect of normalization was found when EMG data were either not normalized, normalized to their own maximum, or normalized to the pre-treatment maximum (van der Krogt et al. 2016). Also, Cappellini (Cappellini et al., 2018), concluded that the choice of the normalization approach had minimal effect.

#### 3.4.3 Data structure

Some data structure methods were used in previous works. Most of the studies documented time normalization before analysis (typically, each stride was resampled to 100 or 101 samples) in order to organize data in matrices before synergy extraction. However, different choices were made on how to organize data structure before synergy extraction. In fact, about half of the papers (53%) concatenated EMG from different strides, as suggested in Oliveira’s work (Oliveira et al., 2014), achieving a “large” matrix that accounts for all the strides. This approach allows to keep track of single-stride variability, by concatenating strides rather than averaging. The authors suggest that concatenation is the best approach to use and this is reflected in the adoption of this method in more than half of the studies. On the contrary, about a third of the papers (31%) extracted synergies directly from single strides. Another approach, used in 16% of the papers, used averaged EMG envelopes before extracting synergies. Details are reported in Figure 4 (upper-right panel).

#### 3.4.4 Algorithms for synergy extraction

The algorithms employed for muscle synergy extraction are reported in Figure 4 (lower-left panel). A vast majority of the studies used non-negative matrix factorization (NMF) (Lee & Seung 1999). In few studies, a variation of NMF was employed to account for missing or corrupted data (weighted NMF or WNMF, Shuman et al., 2018). WNMF preserves the properties of NMF but helps in saving data when they are not recorded properly, which may happen in clinical scenarios. One of the first available paper extracted synergies using an EMG cross correlation approach (Zwaan et al., 2012). The NMF method found wide use as it is frequently employed in the literature and allows to compare findings between works. However, it was used only for the extraction of the spatial synergies, while the dual temporal synergy model implemented with NMF was never adopted. Even if standardization is desirable, some aspects are not uniformly applied (such as convergence criteria and number of replicates), which may lead to slightly different extracted synergies. Moreover, while algorithms such as NMF and WNMF are well established and suit well to clinical scenarios, several other models are available such as spatiotemporal or time-varying synergies (d’Avella and Tresch, 2002), space-by-time synergies (Delis et al., 2014), kinematic-muscular synergies (Scano et al., 2022) that were never applied to these scenarios, and their potential in improving clinical interpretation was not explored. It is believable that these computational approaches may shed light into the mechanisms of motor control that have not been assessed yet in this field. In fact, we believe that exploiting the potential of all the available methods will help to shade light on the neuro-motor control (e.g.,: using spatiotemporal decomposition or space-by-time) and on the functional outcomes occurring due to modified control in which the modifications take place after treatments or surgery. When biomechanics alterations are found after treatments, the kinematic-muscular synergistic approach may allow to detect how biomechanics is related to muscle patterns and the key to the understanding of the modifications rely on the use of synergies in the task space, rather than in their spatial composition.

#### 3.4.5 Selection of the number of synergies

The methods used to select the number of extracted synergies are reported in Figure 4 (lower-right panel). The Variance Accounted For (VAF) threshold approaches are used in many studies and included VAF 90 and VAF 95, used as fixed thresholds for extracting the number of synergies; VAF1 (Steele et al., 2015) shows the amount of variance achieved with one synergy, as an index of the complexity of motor control, also used to compute the walk-DMC (the z-score of the unaccounted variance by a single synergy). The group “Others” includes other VAF-based criteria, including various VAF thresholds (85% and lower, adding further synergies only if they explain a minimum level of variance, 5% or 8% usually), bootstrap-based procedures, or VAFn associated to various orders of factorization. Two studies employed the linear fit technique (Cappellini et al., 2016; Cappellini et al., 2018), and two studies forced *a priori* the number of extracted synergies (Yu et al., 2019; Ettema et al., 2022).

## 4 Discussion

### 4.1 Muscle synergies in physiological locomotion

Human gait can be defined as a series of alternating rhythmic movements of lower extremities that results in forward body progression preserving energy (Perry and Burnfield, 2010). The gait cycle can be divided into 2 main phases: the *stance phase*, starting when the foot touches the ground and the *swing phase*, when the same foot leaves the ground. Most of the works identify four muscle synergies to describe the gait cycle of an healthy adult: the coactivation of the glutei and the quadriceps, in the early stance, guarantees the correct body support; triceps surae assure postural stability and forward propulsion in late stance; the rectus femoris and the tibialis anterior contribute to foot clearance in early and mid-swing, while the hamstrings are involved in leg deceleration in late swing and in the correct leg positioning prior to the subsequent heel strike (Lacquaniti et al., 2012). An additional fifth synergy has been described by some authors as the coactivation of the ileo-psoas and the erector spinae in late stance and early swing (Ivanenko et al., 2004). Since the first studies proving that stroke survivors present a lower number of muscle synergies than healthy controls, an increasing interest has been directed to understand if the same results are observable in DD affecting children (Clark et al., 2010; Cheung et al., 2012).

### 4.2 CP-specific muscle synergies

Most of the studies that investigate how muscle synergies change in children affected by DD focus on CP and generally agree on finding a lower number of muscle synergies with a different activation timing profile when compared to synergies exploited by TD children. In 2020, Bekius published a systematic review on the use of muscle synergy analysis during walking in children with CP (Bekius et al., 2020). The increasing interest in this field is shown by the fact that since then, fourteen more studies have been published on the topic and were deemed eligible for this review. The 31 included studies aimed at understanding how muscle synergies change between CP and TD, in terms of number, spatial composition, temporal recruitment and in terms of correlation with the GMFCS level; if treatments restore healthy-like muscle synergies; and, lastly, which methodological issues need to be resolved by the scientific community, which is one of the point underlined in a recent commentary (D’Avella et al. 2022). Indeed, the most recent works proposed new techniques, including the use of recently released algorithms or multi-modal approaches that may shed light on mechanisms of recovery that have not been fully understood yet. For this reason, in the present review we focused on relevant data processing steps such as normalization and data structure, which are of great interest since the technical methodology may impact the clinical interpretation.

According to most of the studies included in the present review, children affected with CP recruit from two to four muscle synergies during gait. The recruited synergies are either “CP-specific”, eventually resulting from healthy subject’s merging synergies, or “standard synergies”, observed also in healthy controls (Kim et al., 2021). The relative amount of “CP-specific” and “standard” muscle synergies varies in accordance with the severity of the disease. The higher the GMFCS level, the lower is the number of “standard synergies”. The greater the number of “CP-specific synergies”, the poorer the gait performance.

As shown in Table 1, most of the patients affected with CP present the coactivation of the triceps surae during the whole stance and the coactivation of the tibialis anterior with rectus femoris and hamstrings in late stance. The activation of the triceps surae during the whole stance causes ankle plantarflexion and knee flexion as well as hip flexion as a compensatory mechanism to maintain balance, as the center of mass displaces anteriorly. The latter posture is typical of toe walkers (Kelly et al., 1997) The rectus femoris-tibialis anterior couple is not able to provide knee extension and foot clearance for the correct heel strike. The coactivation of the hamstrings together with the physiological synergy composed of the tibialis anterior and the rectus femoris is responsible for the impaired knee extension and the accentuated hip flexion. The earlier activation of the medial hamstrings in late swing and early stance is responsible for the impairment in knee extension. The continuous firing of the rectus femoris causes hip flexion, eventually associated to triceps surae insufficiency typical of crouch gait (Li et al., 2013; Tang et al., 2015; Cappellini et al., 2018; Kim et al., 2018; Yu et al., 2019).

The previously mentioned results are visible as early as a child starts walking, as demonstrated by Dominici et al. (2011) who found that neonates already show patterns of stepping that are retained later (Dominici et al. 2011). Bekius et al. investigated specifically muscle synergies variation before and after the onset of independent walking in both TD and CP children (Bekius et al., 2021). They showed that children with CP recruit less synergies already in supported walking as well as in the first years of independent walking, underlining the need of an early rehabilitation program which might promote an early motor recovery as proposed by Cappellini et al. (2020). Another demonstration of the consistency of muscle synergies relies on finding that CP patients show the same modules among different study sessions, besides a greater stride-to-stride variability when compared to healthy controls (Li et al., 2013; Steele et al., 2015; Goudriaan et al., 2018; Hashiguchi et al., 2018; Yu et al., 2019; Kim et al., 2021).

The presence of different muscle synergies already in the support walking is consistent with the fact that altered gait patters are not the primary cause of changes in muscle synergies, nor the muscle fatigue observed after prolonged walking (Ettema et al., 2022; Spomer et al., 2022). One of the studies included in the review states that the primary cause of crouch gait relies on altered muscle-tendon properties, rather than on reduced neuromuscular control complexity and spasticity (Falisse et al., 2020). On the other hand, the majority of the articles that investigate this issue have reported that in CP toddlers different muscle synergies rely on the abnormal development of brain regions which are responsible for the maturation of the spinal locomotor output and of the locomotor behavior (Cappellini et al., 2018; Bekius et al., 2021). Accordingly, the muscle synergies result to be mined since the first steps of the child and they remain rather consistent throughout life, being the result of a central nervous system impairment (Cappellini et al., 2016; Bekius et al., 2020; Short et al., 2020).

### 4.3 The effect of treatments on CP-specific muscle synergies

Few papers investigate the effects of treatments on children with CP: only 4 out of the 31 included articles address the topic. The limited number of studies limit the interpretation of how treatments may intervene in reshaping muscle synergies. However, some common lines are accepted. First, a general consensus has been reached in finding amelioration in gait pattern following treatments, particularly in children with GMFCS level I and II. However, while biomechanics and kinematics improve, none of the studies have proved clear changes in muscle synergies weighting, while they have proved limited effects regarding temporal activation (Shuman et al., 2019). The currently described non-alteration in spatial synergies can be justified according to the most accepted findings at the moment: muscle synergies are encoded before the child acquires the ability to walk (Dominici et al., 2011; Bekius et al., 2021). When the child starts walking, the previously occurred brain lesions impair the neuromuscular control system, resulting into faulty muscle recruitment (Cappellini et al., 2018; Bekius et al., 2021). Orthopedic treatments generally occur later in time and, indeed, they eventually improve gait patterns, without modifying synergies, encoded long before (Shuman et al., 2018; Oudenhoven et al., 2019; Shuman et al., 2019). Noteworthy, some authors have shown that children with CP can selectively adapt and improve aspects of gait when challenged with bio feedbacks (Booth et al., 2019; Cappellini et al., 2020) as well as they show worse performance when challenged with environmental changes (Kim et al., 2022).

Interestingly, a slightly higher number of studies have employed muscle synergies for assessing rehabilitation protocols of upper limbs, especially in stroke patients, showing that the number and spatial structure of synergies eventually rarely change in these patients following treatments. Consequently, as already proposed in a previous review (Bekius et al., 2020), the temporal structure of synergies could be a target for treating lower limb impairments in CP patients. Furthermore, we would like to extend this concept and propose that the space related performance achievable with each synergy could be a training target too. Assuming that no changes occur following treatment, the same pre-treatment synergies can be recruited with different timings or, more precisely, with a different biomechanical function. This assessment can be achieved with correlation analysis, factor analysis, or with the Mixed-Matrix Factorization (MMF) algorithm (Scano et al., 2022).

### 4.4 How muscle synergies vary in other developmental diseases

The choice of including other neurodevelopmental diseases than CP was associated to the need of better understanding the etiology of the variation of muscle synergies in children affected by DD. According to what observed so far in CP, the CP specific muscle synergies potentially result from brain lesions. If this is the case, we expected not to find any differences in other pathologies which do not directly mine the nervous system.

Two of the articles included in the present review analyze muscle synergies in children affected by Duchenne muscular dystrophy. The absence of the protein dystrophin is responsible for the infiltration of the muscular tissue with non-contractile fibrofatty tissue leading to muscular weakness. While weakness in CP has neural and non-neural components, in DMD weakness can be considered as a predominantly non-neural problem. As in CP, non-neural muscle weakness has little influence on complexity of motor control during gait. Both studies agreed on not finding any differences in synergy weights and activation within respect to healthy controls (Goudriaan et al., 2018; Vandekerckhove et al., 2020).

Other studies have focused on how muscle synergies change in different pathologies ranging from Down syndrome and spinal cord injuries to hemophilic arthropathy.

Three groups of authors have respectively investigated the role of muscle synergy analysis in intellectual disability, incomplete spinal cord injuries and Down syndrome. People with intellectual disability exploit the same muscle synergies as healthy peers during motor tasks, but they have limited ability to use somatosensory information and to adapts their postural muscle responses to repeated external perturbation, which is consistent with what observed in children exposed to acute painful stimuli (Blomqvist et al., 2014; Van den Hoorn et al., 2015). Children with incomplete spinal cord injuries most often rely on synergistic muscle coactivation across the flexion and extension phases of different motor tasks, presenting different muscle synergies when compared to healthy peers (Fox et al., 2013).

Lastly, the paper on the emergence of neuromuscular patterns during walking in toddlers affected by Down syndrome and typically developed toddlers demonstrated that a period of 6 months of practice is needed to acquire a rhythmic and stable muscle activation pattern during independent walking. Still, following the first 6 months, typically developed toddlers present efficient synergies among muscles, allowing increased relaxation time between muscular bursts, while toddlers affected by Down disease present inconsistent timing of muscular activation (Chang et al., 2009).

Another study on hemophilic arthropathy demonstrated that the disease reduces the complexity of the neuromuscular control, probably due to chronic painful stimuli (Cruz-Montecinos et al., 2019). Differently, acute noxious stimulations did not show any changes in motor control **(** Van den Hoorn et al., 2015). In the previously two mentioned cases, the different afferent information might cause an impaired neuromuscular response. In both cases the problem occurs when the child has already started walking, experiencing the four muscle synergies that underlie locomotion. We believe that the reason why this happens is because chronic pain might cause a plastic rearrangement in the corticospinal tract up to select muscles differently. On the other hand, acute pain does not allow a plastic rearrangement.

### 4.5 Muscle synergy extraction methods

Pipelines for muscle synergy extraction employed in previous studies require several steps for analysis. Typically, these steps include preprocessing (filtering and aligning), segmentation, normalization (in time and on EMG amplitude), organization of the data in a specific data structure before synergy extraction (in a compatible way with the factorization algorithm), synergy extraction, implementation of the algorithm for comparison between extracted synergies to address the research questions. For the aims of this paper, a systematic revision of five steps has been made: pre-processing, data normalization, data structure, algorithms for synergy extraction, and algorithms for determining the number of synergies to extract.

EMG preprocessing is a crucial step needed before synergies are extracted. In particular, the frequencies adopted in literature for the low-pass filtering fall in a wide range (from 4 to 40 Hz as reported already in Shuman et al., 2017). When changing the cut-off frequency, not only EMG envelopes show different shapes, but also the VAF curve are modified and the number of extracted synergies may be misinterpreted. Setting specific guidelines, as it was done for other fields of research in which EMG analysis is performed, would increase inter-study comparison and data operability. Some adaptions proposed include the adaption of the filtering low-pass frequency according to the velocity (Hug et al., 2012), but this suggestion was rarely adopted in further studies.

EMG amplitude normalization is needed to allow inter-subject and inter-session comparison, and to account for reduced signals coming from muscles having lower volume and activation that would explain very little variance if EMG data were not normalized. In fact, lower EMG signals may be due to intrinsic features inherent to that muscle (number of motor units activated; detection area; muscle geometry, and others) rather than muscle non-use. Interestingly, it was found that non-uniform approaches for normalization were employed in literature. In more than half of the studies, researchers normalized each EMG activity with respect to the maximum EMG value found in all trials for that session. This approach seems the most similar in measuring MVC, which cannot be applied to these scenarios as MVC cannot be easily and reliably measured. However, other approaches were used in several works, including single-stride normalization, normalization to achieve unit variance on each channel, and others. The issue of which procedures should be adopted for normalization is an open question in muscle synergy analysis and it may constitute a source of bias for inter-study comparison, as well as a limiting factor for generalizing results. Few papers considered this issue and verified whether normalization may alter results (van der Krogt et al., 2016; Cappellini et al., 2018). There is a general agreement in stating that the choice of the normalization approach has a minimal effect, probably due to the fact that tests on patients include generally a limited number of conditions. Shuman and others suggested that some approaches based on z-scores may reduce the effect of normalization (Shuman et al., 2017). However, these conclusions cannot be generalized to other studies and a uniform choice of EMG normalization is one of the gaps to fill for generating optimal and commonly accepted guidelines for inter-study comparisons. We also noted that while the normalization itself may account only for slight differences, its “cascade” effects when non-uniform approaches are used for filtering, data structure, etc. might become relevant. Novel approaches available for the field such as the recently released MMF will pose further questions for researchers, such as how to filter and normalize data coming from different domains when they are factorized together (such as the case of kinematics and EMG).

Similarly, data structure used for extraction show relevant differences between studies. In nearly half of the studies (53%), data from various strides were concatenated. This approach preserves the variability between steps, but may capture noise-related features of the movement. According to Oliveira’s work (Oliveira et al. 2014), concatenating is the best method for structuring data in locomotion. About a third of the studies extracted synergies from single strides instead. While this process captures inter-stride variability, it may also interpolate noise and generate noise-driven synergies (D’Avella et al., 2022), thus misinterpreting the synergies underlying gait, especially when very few steps are available. Lastly, in a minor number of papers, data were averaged. This approach allows to remove noise, but loses sensibility in quantifying single stride variability. It is worthy to note that the choice of the approach impacts on VAF curves; thus, it impacts most of the VAF-based metrics used to select the number of synergies.

The results reported in our screening refer mainly to the NMF algorithm and its variation WNMF, which are the state-of-the-art for synergy extraction (especially in this field). Regarding the employed algorithms so far, the screened studies are uniform. Non-negative matrix factorization is considered the most appropriate method for extraction of muscle synergies in walking and running (Rabbi et al., 2022) and all the studies (except one) employed it (or its variation WNMF) to extract synergies. This can be now considered as a consolidated standard, allowing fair comparison of the results between studies. However, this uniform and use of NMF and WNMF so far can be seen also as an opportunity. In fact, other algorithmic approaches can be used in future work and they might open a variety of novel insights. It was already suggested that spatial-temporal synergies may relate better with the patients’ clinical functioning level (d’Avella et al., 2022); the time-varying algorithm might help in showing subtle changes which might not be detected with the standard NMF, as it was already found in studies regarding the upper-limb (Berger et al., 2020). Considering kinematic and EMG waveforms together as proposed with the mixed matrix factorization (Scano et al., 2022) might improve the interpretation of the data and provide links between muscle synergies and their motor outcome, thus providing further elements to verify how stable synergies can be related to improved motor outcome after treatments. In fact, considering the little modifications of synergies found in pathology, the MMF algorithm opens the way to the study of biomechanics or kinematics and muscle synergies in the same factorization, providing novel keys of interpretation encompassing both motor coordination and functional outcomes. Lastly, the use of temporal models, rarely employed in this field of research, could enhance the comprehension of the mechanisms connected to temporal recruitment of coordinated muscles. The development and the application of novel algorithms might help in understanding the neurophysiological mechanisms underlying the disease and are probably one of the main ways to pursue future researches. We also suggest that clinical applications are probably not following the rate at which novel methods are made available, missing promising directions and valuable tools toward the development of the field.

While the choice of the number of synergies is a further source of uncertainty, the number of synergies for walking are well-established and determined in physiological gait. However, when pathological gait is analyzed, the number of synergies might be altered and thus also the determination of the number of synergies should be standardized in a definitive way. Most of the screened studies employed VAF threshold algorithms, with quite consistent threshold levels adopted between studies, often coupled with the VAF1 criterium that measures the complexity of motor control. However, according to previous literature, the linear fit model was suggested as a valid alternative method for determining the appropriate number of synergies (Borzelli et al., 2013), as it is potentially less sensitive to VAF shifting found when changing the filtering parameters. To determine the minimum number of basic activity patterns *n* that best accounts for the EMG data variance, the best linear fit is based on a linear regression procedure (D’Avella et al. 2006) by varying the number of basic patterns from 1 to 8 and selecting the smallest *n* such that a linear fit of the VAF vs. *n* curve had a residual mean square below a specific threshold. The performance of the linear fit for this aim should be tested more.

Methods for further analysis (after synergy extraction) are very study-specific and are not reported in detail since they depend heavily on the purpose of the study and cannot be compared systematically. However, future work should also focus on how synergy analysis methods could be used to maximize the interpretation of the extracted synergies.

Lastly, recent studies in muscle synergy analysis from other fields suggest to repeat the selection of the number of synergies according to multiple criteria to confirm the solidity of the results, for example, by adopting multiple VAF thresholds (Pale et al., 2020). This approach might be meaningful especially when selecting the number of synergies for patients.

### 4.6 Future directions for the clinical application of muscle synergies

A growing interest in muscle synergy analysis in DD has been demonstrated with a growing number of published papers. In 2022, 7 articles were published on muscle synergy analysis in CP and since the last systematic review conducted by Bekius and others (Bekius et al., 2020), fourteen publicly studies have become available on the main databases. The novel works brough to light some important questions which are still open, as the optimization of the extraction methods and the prediction of the effects of rehabilitation treatments on muscle synergies. This review offers a summary on the currently open points in the field and compare the aims, the methodology and the results of the study conducted so far, with all the technical aspects in terms of data extraction.

In the following paragraphs, we summarize some of the main points that we believe will be considered for a more effective application of the method in the future, to exploit its potential and foster a systematic use of muscle synergies in clinical practice.

#### 4.6.1 The selection of the study population

The choice of the best experimental design and methods relies not only on applying the most suitable synergy extraction model, but also on selecting the most suitable study population, in terms of inclusion-exclusion criteria, matching and grouping.

One of the main exclusion criteria observed in most of the study protocols are previous surgeries. Still, none of the studies have proved changes in muscle synergies weighting and temporal activation following treatment (Shuman et al., 2018; Oudenhoven et al., 2019; Shuman et al., 2019). We see in this discrepancy an opportunity to better understand the role of previous surgeries in the study design. One of the points that needs to be clarified is if the GMCSF level does not change at all besides the already proved post-surgical biomechanical amelioration. This point might help in understating if surgery is indeed a confounding factor as it is considered now and which surgical interventions, ranging from tendon transfers to injections, must be eventually considered as confounding factors.

Apart from surgeries, we have noticed a high level of variance in the criteria of selecting the study population, even among the most refined studies. Goudriaan focused on the gait patterns (Goudriaan et al., 2022), Bekius matched cases and control based on the walking abilities (Bekius et al., 2021), whereas Kim et al. decided to include only unilateral CP patients (Kim et al., 2018; Kim et al., 2021). All the previous aspects are of great importance; the possibility of studying muscle synergies according to the gait patterns (Goudriaan et al., 2022; Spomer et al., 2022) might be a starting point to consider customized rehabilitation protocols, as it is the case for studying only unilateral CP patients (Kim et al., 2021). Still, even if the high variance among the study designs is of great importance in offering different points of view, it makes harder to compare the clinical results.

Some considerations can be made on the selection of cases and controls. Besides the selection protocol, the case-control matching is generally based on the age of the patients, and then cases are further subdivided according to the GMFCS level. The subdivisions of the patients according to the severity of the disease has demonstrated a direct correlation between the GMFCS level and the walking impairment which strengthens the clinical role of the GMFCS scale by adding to it a neuro-engineering based value, represented by muscle synergies. This concept is especially true for GMFCS level I and II which indeed present a lower number of CP-specific muscle synergies compared to GMFCS III and IV (Yu et al., 2019).

On the other hand, we believe that the age of the patient is one of the interesting fields that should be investigated more in depth. According to Bekius and others muscle synergies do not change before and after the onset of independent walking (Bekius et al., 2021). These results are coherent with the fact that CP is caused by a non-progressive brain lesion and with the thesis that muscles synergies impairments are mainly due to brain lesion occurring very early in life (Cappellini et al., 2020; Short et al., 2020). We think that it would be of interest to evaluate a same cohort of patients at different ages to assess if any changes occur as the patient gets older and therefore, if there is a more suitable time for treatments.

#### 4.6.2 The acquisition session

A limiting factor to the systematic adoption of synergies in clinical practice is the time-consuming procedures needed for acquiring data and the “transparency” of the equipment and apparatus when worn by patients. An interesting approach to simplify synergy extraction protocols and to ease the adoption of synergies has been offered by Rabbi et al. who have proposed a new model of extracting muscle synergies based on limiting to the minimum the number of the analyzed muscles in CP children and to reconstruct the EMG signal of the missing muscles from what is observed in TD children, thus increasing the clinical applicability of the employed methods. This approach reduces the time needed for data acquisition and therefore patients’ stress, making muscle synergy analysis more applicable also in clinical practice (Rabbi et al., 2022). Besides the need of analyzing more subjects to validate the previously mentioned method, we believe that reducing the number of probes is a promising approach, even if there are some muscles that must be always recorded in CP patients, being the ones forming the CP-specific synergies, as the tibialis anterior, rectus femoris, medial hamstrings and gastrocnemii.

Furthermore, even if some muscles are considered more meaningful and therefore they are more used among the studies, we need to bear in mind that the number of muscles and the muscles analyzed can interfere with the results. The same is true for the number of strides that should be high enough to make normalization choices meaningful, and in order not to capture trial specific features rather than synergies, as well as to deepen the analysis on temporal components (Bekius et al., 2020 **)** that may benefit from the availability of multiple strides. For all these reasons, we must be careful in choosing the right minimum number of muscles as well as the right minimum number of strides.

#### 4.6.3 The role of muscle synergies in clinical practice

We believe that the great push fostering the application of muscle synergy analysis in clinical practice does not aim only at diagnosing and describing quantitatively the disease, but also at understanding the underlying neurophysiological mechanism for offering the best therapeutical option.

The latest studies have tried to understand both the etiology of muscle synergy variation and the effect of treatments on muscle synergies and biomechanics.

According to Spomer and others, children emulating CP gait exploit neither the same synergies of CP children nor the usual synergies, proving that biomechanics is not the unique cause of altered motor performance (Spomer et al., 2022). The role of biomechanics has been investigated by Falisse et al. who identified few causes of CP motor impairments as control impairments, spasticity, bone deformities and altered tendon properties (Falisse et al., 2020). Muscle fatigue is not involved in muscle synergy changes as proved by studies on CP as well as on Duchenne muscular dystrophy (Goudriaan et al., 2018; Vandekerckhove et al., 2020; Ettema et al., 2022), whereas the cortex plays a crucial role in determining altered muscle synergies, as demonstrated by Cappellini et al. (2020). As follow, we can agree on defining CP as a disease with a non-univocal etiology, at least for what it is known so far.

Considering synergy extraction protocols, non-negative matrix factorization can be considered as a consolidated standard, allowing comparison of the results between studies. However, the perspective from the standard spatial synergy model already explored cannot probably fully explain the mechanisms underlying the pathology. Algorithms “free” from non-negative input constrains such as MMF for kinematic-muscular synergies (Scano et al., 2022) have not been employed yet and may shed light on the neurophysiological mechanisms underlying pathology, and might help in correlating gait patterns, GMFCS level and muscle synergies. Such models foster multi-modal approaches: they include spatio-temporal features and allow to link muscular variables to the outcome variables. Moreover, d’Avella and others have recently focused on the current pitfalls and challenges in synergies extraction that should be addressed in future work (d’Avella et al., 2022). They include the avoidance of extracting noise-dependent synergies, matching pairs of temporal or weighting activation profiles at their best, discriminating power dependence on the type of synergistic model. Novel directions focus also on improving the knowledge of the mechanisms underlying the pathology: it has been recommended that techniques such as magnetic resonance imaging should be employed to provide a more discriminative representations of individual motor control strategy (d’Avella et al., 2022). Such issues should be solved with the purpose of trying to extract task-specific features of motor variability affecting performances and to correlate synergies with anatomopathological information (D’Avella et al., 2022).

Four of the thirty-one studies on CP focus on treatment outcomes. Even if Spomer showed that biomechanics is not the unique cause of altered motor performance in CP patients (Spomer et al., 2022), it is also true that, according to the published studies, biomechanics is the main aspect that changes in the post treatment period (Shuman et al., 2018; Oudenhoven et al., 2019; Shuman et al., 2019). A more refined analysis on temporal components (Bekius et al., 2020) may also clarify better whether synergies are not modified, but still used in new ways after treatment. Again, four articles are a limited number to make any conclusions on what happens following treatment, which constitutes one of the main directions for future research. Some relevant models such as the spatio-temporal one (d’Avella and Tresch, 2002) might find subtle differences among pretreatment and post treatment synergies, which cannot be revealed by the NMF model, which does not capture time-varying features.

Lastly, since it remains controversial how muscle synergies and clinical observation convey the same information on motor impairment, it is possible to work more on multi-modal approaches by linking features from the synergy domain to items of clinical scales, as recently proposed for upper-limb investigations by combining factor analysis with linear regression methods (Maistrello et al., 2021). These approaches represent a promising way to integrate clinical assessments and synergies and to understand how they relate.

#### 4.6.4 The role of muscle synergies in other developmental diseases

According to what observed in literature, the application of muscle synergies in developmental diseases other than CP did not show any differences in cases and controls in DMD (Goudriaan et al., 2018; Vandekerckhove et al., 2020) and in intellectual disability (Blomqvist et al., 2014). Few differences were observed in chronic painful stimuli, as in hemophilic arthropathy (Cruz-Montecinos et al., 2019), while no changes were spotted in acute noxious stimuli (Van den Hoorn et al., 2015). The possibility of analyzing other pathologies in the present review helped in better understanding the etiology of altered muscle synergies in patients. DMD is characterized by a peripheral weakness which apparently does not interfere with the recruitment of muscles by the CNS. On the other hand, chronic pain might cause a plastic rearrangement in the corticospinal tract up to cause a selection of different muscles. According to this, we believe that it might be less useful applying muscle synergies in those pathologies in which there is not an impairment and/or rearrangement of the motor control at a central level, as it happens in CP, which is mainly caused by cortical and subcortical lesions, and in chronic pain, which causes neurological plastic rearrangement.

## 5 Conclusion

The current review of literature demonstrates that there is an increasing interest in applying muscle synergy analysis in children affected by developmental diseases. The scientific community aims at understanding how the neuromotor control is impaired in developmental diseases, with a special interest in CP. Indeed, the main changes in muscle synergies appear to have a neurological origin and treatments appear to influence gait biomechanics, but to have very little effects on muscle synergies. All the studies included in the review agree on finding a lower number of muscle synergies in CP children during locomotion. They also show an indirect correlation between the number of synergies and the GMFCS level. In Duchenne muscular dystrophy the weakness has a non-neural origin and, accordingly, no changes in muscle synergies were observed. On the other hand, patient affected by incomplete spinal cord injuries present fewer muscle synergies than healthy peers, as it is for children affected by intellectual disability and by chronic painful condition, possibly due to plastic changes in response to the afferent information conveyed to the cortex.

There are many aspects that must be still determined: the application of new, potentially useful protocols that are based on synergies, also for clinical decision making; the focus on different aged population to better understand if there is a perfect timing for treatment; the design of longer follow-up studies which might highlight changes as a result of the plasticity of the CNS.

Even though many scientific results have been achieved, the neurophysiological mechanism of DD, particularly of the CP, are still not completely known, and there is the need to translate the researches’ results to provide the best clinical care for DD patients.
